# Overexpression of a *Hevea brasiliensis ErbB-3 Binding protein 1* Gene Increases Drought Tolerance and Organ Size in *Arabidopsis*

**DOI:** 10.3389/fpls.2016.01703

**Published:** 2016-11-14

**Authors:** Han Cheng, Xiang Chen, Jianshun Zhu, Huasun Huang

**Affiliations:** Key Laboratory of Rubber Biology, Ministry of Agriculture, Rubber Research Institute, Chinese Academy of Tropical Agricultural ScienceDanzhou, China

**Keywords:** drought stress, *Hevea brasiliensis*, ErbB-3 Binding Protein 1, *Arabidopsis*, organ size, cell cycle

## Abstract

Rubber trees are economically important tropical tree species and the major source of natural rubber, which is an essential industrial material. This tropical perennial tree is susceptible to cold stress and other abiotic stresses, especially in the marginal northern tropics. Recent years, the genome sequencing and RNA-seq projects produced huge amount of sequence data, which greatly facilitated the functional genomics study. However, the characterization of individual functional gene is in urgent demands, especially for those involved in stress resistance. Here we identified and characterized the rubber tree gene *ErbB-3 binding protein 1*, which undergoes changes in expression in response to cold, drought stress and ABA treatment. *HbEBP1* overexpression (OE) in *Arabidopsis* increased organ size, facilitated root growth and increased adult leaf number by delaying the vegetative-to-reproductive transition. In addition, *HbEBP1* OE enhanced the resistance of the *Arabidopsis* plants to freezing and drought stress, demonstrating that this gene participates in the regulation of abiotic stress resistance. *RD29a, RD22* and *CYCD3;1* expression was also greatly enhanced by *HbEBP1* OE, which explains its regulatory roles in organ size and stress resistance. The regulation of drought stress resistance is a novel function identified in plant *EBP1* genes, which expands our understanding of the roles of *EBP1* gene in response to the environment. Our results provide information that may lead to the use of *HbEBP1* in genetically engineered crops to increase both biomass and abiotic stress resistance.

## Introduction

ErbB-3 Binding Protein 1 is a member of the proliferation-associated 2G4 protein (PA2G4) family (Lamartine et al., 1997; Lessor et al., 2000; Xia et al., 2001; Squatrito et al., 2004). This recently identified transcription factor is involved in multiple pathways such as the cell cycle, protein translation, rRNA synthesis and cell proliferation (Squatrito et al., 2006; Sun et al., 2012; Figeac et al., 2014; Liu et al., 2014), although detailed mechanisms for each of these activities have yet to be determined. In humans, HsEBP1 binds dsRNA to form part of the ribonucleoproein (RNP) complexes via association with different rRNA species (Squatrito et al., 2004). EBP1 also associates with mature ribosomes and suppresses the phosphorylation of the eukaryotic initiation factor 2 alpha under stress condition, and thus is possibly involved in the control of protein translation (Squatrito et al., 2006). In T cells, EBP1 is required for the regulation of ribosomal RNA synthesis (Nguyen et al., 2015). EBP1 also functions as a nuclear cell survival factor that interacts with Akt and PKC to inhibit apoptosis (Ahn et al., 2006).

EBP1 encodes two isoforms, p48 and p42; p48 enhances cell growth, whereas p42 stimulates cell proliferation (Liu et al., 2006). Ectopic expression of EBP1, however, inhibits proliferation and induces differentiation in the breast carcinoma cell lines AU565 (Lessor et al., 2000). EBP1 is also up-regulated in the sciatic nerve after crushing of the nerve, where it is believed to be involved in the differentiation and migration of Schwann cells (Liu et al., 2014). Recently, EBP1 was reported to bind Anxa2 and negatively regulate the proliferation and invasion of breast cancer cells (Zhang et al., 2015). Knockdown of *EBP1* inhibits both the proliferation and differentiation of resident stem cells of skeletal muscle, which cannot be rescued by ErbB3 over-expression, suggesting that EBP1 controls the proliferation and differentiation of muscle stem cells (Figeac et al., 2014).

EBP1 is also functionally conserved in plants. The expression of plant *EBP1* is tightly regulated and is remarkably correlated with organ growth in a dose-dependent manner (Horváth et al., 2006). Plant *EBP1* is also required for the expression of *CyclinD3;1, ribonucleotide reductase 2* and the *cyclin-dependent kinase B1;1* genes and thus is involved in cell cycle regulation, as shown in *Solanum tuberosum* and *Arabidopsis thaliana* (Horváth et al., 2006). *EBP1* from *Ammopiptanthus mongolicus* is up-regulated by cold, and it’s OE in *Escherichia coli* and *Arabidopsis* confers cold and freezing tolerance by accelerating ribosome biogenesis and the translation of transcriptional factors and downstream functional proteins that are induced under cold stress (Cao et al., 2008). In maize, *ZmEBP1* gene has an overdominant expression pattern in the immature ears of hybrids that exhibit heterosis (Wang et al., 2016). The OE of *ZmEBP1* in *Arabidopsis* increases organ size by accelerating cell proliferation. Thus the *EBP1* genes have conserved functions across eukaryotes.

Rubber trees (*Hevea brasiliensis*) are tropical perennial trees that are susceptible to cold stress (Cheng et al., 2015). In China and other regions in the northern edge of the tropics, rubber tree plantations suffer from cold stress in the winter. Although some cold-resistant clones have been bred in China, there is an urgent demand for additional clones with stronger cold resistance. The development of transgenic technique in rubber trees may help to meet this demand more quickly (Montoro et al., 2003). Although the high-quality rubber tree genome was recently released (Tang et al., 2016), the functional genes that related to stress resistance are still to be identified individually (Cheng et al., 2015). Previously, we constructed a cold- induced full-length cDNA library (Cheng et al., 2008), in which *HbEBP1* was highly abundant. Further characterization revealed that *HbEBP1* responded to cold and drought stress and ABA treatment. Here we characterized *HbEBP1* and overexpressed it in *Arabidopsis*, which showed that this gene regulates organ size and resistance to drought and cold stresses in rubber trees.

## Materials and Methods

### Plant Material, Preparation of Transgenic Plants

The 1-year-old rubber tree clone 93–114 was maintained as described (Cheng et al., 2015). *Arabidopsis thaliana* ecotype Columbia-0 (*Col-0)* was used as wild-type in this study. Seeds were surface sterilized and sowed in pots (10 cm × 10 cm × 10 cm) containing a mixture of soil and vermiculite (3:1 v/v). After 4 days imbibitions at 4°C, the plates and pots were transferred to a growth chamber at a constant temperature of 20°C under 16 h light (125 μmol m^-2^ s^-1^)/8 h dark cycles.

To generate the *Arabidopsis* plants that overexpress *HbEBP1* (*HbEBP1* OE), the coding region of *HbEBP1* was amplified using primers 5′-TCAAAGCTGTAAGCTTATGTCGG-3′ and 5′-CATAAGAATTCCATACAAGGT-3′. The coding sequence fragment was then subcloned between the *Hin*dIII and *Eco*RI sites in pXCS-HAStrep plasmid. *Arabidopsis* ecotype *Col-0* plants were transformed according to the floral dip method (Clough and Bent, 1998) using *Agrobacterium tumefaciens* strain GV3101(*pMP90RK*).

### Manipulation of DNA, RNA Isolation, and Expression Analysis

Isolation of rubber tree DNA and RNA was carried out as described (Zewei An, 2012). For northern blotting, a *HbEBP1* probe was prepared from full-length cDNA with DIG Northern Starter Kit (Roche, USA). Twenty micrograms of total RNA was run on a denature 1.5% agarose gel and the blot was then transferred onto an Amersham Hybond^TM^-N^+^ nylon membrane (GE Healthcare, USA). Hybridization and detection were carried out according to the manufacturer’s instructions.

Total RNA was extracted from *Arabidopsis* seedlings using Qiagen RNeasy Plant Mini Kit (Qiagen, USA). For quantitative RT-PCR (Q-PCR) analysis, the RNA samples were treated with DNase I to remove possible DNA contamination. Two micrograms of total RNA was used for each detection. The first-strand cDNA was synthesized with SuperScript III according to the manufacturer’s instructions (Invitrogen, USA). Q-PCR was carried out using IQ SYBR Green Supermix (Bio-Rad, USA) with gene-specific primers as listed in Supplemental Table S1. PCR was performed on a Bio-Rad Iq5 RT PCR instrument using the following program: 95°C for 3 min, followed by 40 cycles of 95°C for 15 s, 60°C for 30 s and 72°C for 30 s.

### Bioinformatic and Phylogenetic Analysis

Multiple sequence alignments were performed using ClustalW2 at the EBI ClustalW server^1^ using the default parameters (Larkin et al., 2007). The coding sequence was predicted using Bioedit software and confirmed by using BLASTP program at NCBI BLAST server^2^. The protein secondary domains were predicted with Interproscan program (Jones et al., 2014) at EBI server^3^. MEGA software (version 6.06) was used for phylogenetic analysis (Tamura et al., 2013). A phylogenetic tree was constructed using the neighbor-joining method with bootstrap test. The default parameters were used for the construction.

### Southern Blotting

Southern blotting analysis was carried out as described (Cheng et al., 2015). Briefly, 20 μg *Arabidopsis* genomic DNA was digested with *Eco*RI. The DNA was resolved on a 0.8% agarose gel and transferred onto an Amersham Hybond^TM^-N^+^ nylon membrane (GE Healthcare, USA); the blot was cross-linked under 0.12 J/cm^2^ UV irradiation. Then the blot was hybridized at 42°C with Digoxigenin-labeled probe for 12 h. After two stringent washes at 68°C, the signal was detected using DIG Nucleic Acid Detection Kit (Roche, USA).

### Cold and Drought Stress and ABA Treatment

Cold and drought stress and ABA treatment in rubber trees were carried out as described (Cheng et al., 2015). To measure cold resistance in *Arabidopsis*, an electrolyte leakage analysis was performed as described (Cheng et al., 2013). Briefly, electrolyte leakage was measured using leaves from 2–week-old seedlings that had been frozen to -7°C at a cooling rate of 1°C/h from -1°C with an A28F Thermo Fisher temperature-controlled water bath (Thermo Scientific, USA).

To calculate dehydration rate, leaves were detached from 2-week-old seedlings, weighed and placed on dry filter paper in a drying chamber. The samples were weighed at 30-min intervals and the dehydration rate was calculated as the ratio of the remaining weight to the initial weight at each time point.

The drought tolerance test was carried out by sowing seeds in pots containing vermiculite. Irrigation was performed daily by adding half Hoagland nutrient solution in the tray until all the liquid was absorbed. When the seedlings were 2 weeks old, irrigation was withheld for 1 week. At least three pots of seedlings were observed for each genotype in this test.

For cold treatment in rubber tree, the seedlings were transferred to a 4°C cold culture room. Drought was carried out by detaching the leaf from the seedlings and keeping on the filter paper at room temperature. For ABA treatment, 100 μmol/L ABA solution was sprayed onto the seedlings until the liquid dropped from the leaf. Then the seedlings were covered with a plastic bag. The leaf sample was collected at indicated time points and stored in liquid nitrogen immediately.

### Developmental Phenotypic Analyses

The leaf development phases were determined by observing abaxial trichome production as described (Willmann and Poethig, 2011). The emergence of rosette leaves was recorded every day from day 7 after planting based on the criteria that they could be recognized with the naked eyes. Flowering time was recorded as the day on which flower primordia were visible without the aid of a microscope. Leaf length and blade length and width were measured as described (Willmann and Poethig, 2011). For leaf area measurements, the leaves were carefully detached from the seedlings and pasted on a sheet of A4 paper and then were scanned. The images were then analyzed with Image J software (version 1.45s) to calculate leaf area (Schneider et al., 2012).

For root length measurement, seeds were surface sterilized and sown onto the surface of a square plate containing half MS agar medium, and were then cultured vertically. Photos were taken daily, and root length on each day was measured with a ruler. At least 20 individual seedlings were analyzed from each line in these studies.

For measurement of the leaf epidermal cells, ten *Arabidopsis* plants were selected randomly. The area of the fully expanded seventh leaf was measured at about 25 days after germination in each transgenic line and *Col-0* plants. The detail measurement was performed according to the method reported (Wang et al., 2016). Total cell number per leaf was calculated by dividing the leaf area by the average cell size.

### Statistical Analysis

All results are presented as the mean ± standard error of the mean (SEM) from five biological replicates for the stress experiments or from the number of replicates indicated. Statistical analysis was performed by using the Student’s *t*-test, and *p* < 0.05 was recognized as significant.

## Results

### Characterization of *HbEBP1*

**FIGURE 1:**
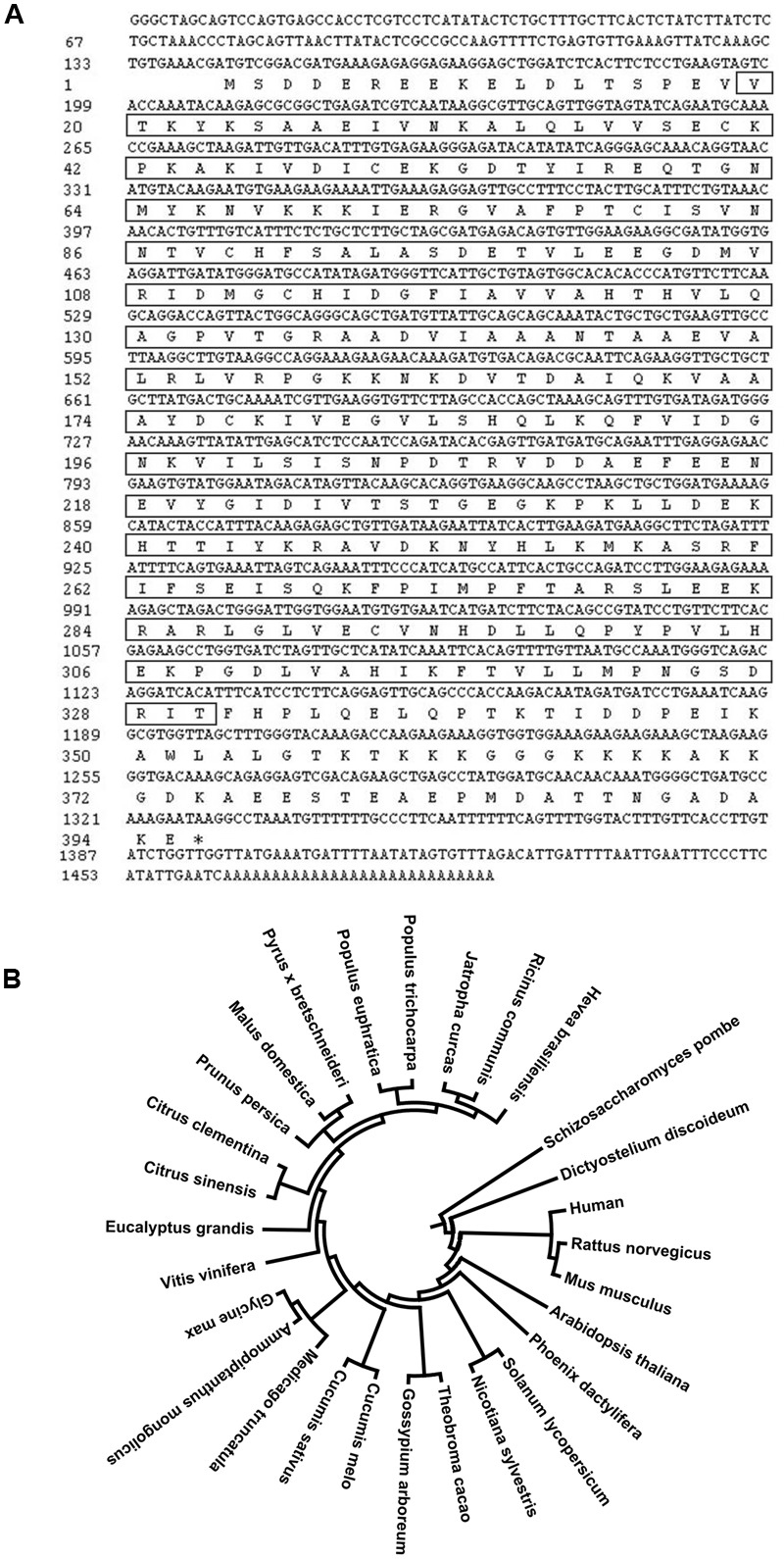
**(A)** Full-length cDNA sequence of rubber tree *ErbB-3 binding protein 1* (*HbEBP1*) and its deduced encoding peptide. The sequence in the square frame corresponds to the deduced MAP domain. **(B)** Phylogenetic tree showing similarity of the deduced *H. brasiliensis* EBP1 protein sequence with EBP1s from other plant species. EBP1 peptide sequences of each species were downloaded from GenBank based on the following accession numbers: *Ricinus communis*, XP_002530504.1; *Jatropha curcas*, XP_012089820.1; *Populus trichocarpa*, XP_002308228.2; *Populus euphratica*, XP_011046861.1; *Pyrus* × *bretschneideri*, XP_009376423.1; *Malus domestica*, XP_008362268.1; *Prunus persica*, XP_007205303.1; *Citrus clementina*, XP_006429434.1; *Citrus sinensis*, KDO56711.1; *Eucalyptus grandis*, XP_010037691.1; *Vitis vinifera*, XP_002262986.2; *Glycine max*, XP_003529167.1; *Ammopiptanthus mongolicus*, ABF66654.1; *Medicago truncatula*, XP_003623305.1; *Cucumis melo*, XP_008442393.1; *Cucumis sativus*, XP_004137704.1; *Gossypium arboreum*, KHG25370.1; *Theobroma cacao*, XP_007026646.1; *Nicotiana sylvestris*, XP_009789408.1; *Solanum lycopersicum*, XP_004242516.1; *Phoenix dactylifera*, XP_008805004.1; *Arabidopsis thaliana*, Q96327.1; *Mus musculus*, P50580.3; *Rattus norvegicus*, Q6AYD3.1; *Homo sapiens*, Q9UQ80.3; *Dictyostelium discoideum*, Q1ZXG4.1; *Schizosaccharomyces pombe*, Q09184.1. The phylogenetic tree was constructed using a MEGA 6.06 software.

We previously identified a cDNA clone that putatively encodes a proliferation-associated 2G4 family member from the cold induced full-length cDNA library (Cheng et al., 2008). This cDNA fragment is 1462 bp in length, and contains an 1188 bp coding region (**Figure 1A**). The predicted peptide is highly similar to EBP1 proteins in other plant species and was therefore designated as HbEBP1. The deduced HbEBP1 amino acid sequence contains a proliferation-associated PA2G4-like domain, an aminopeptidase APP_MetAP domain and a methionine aminopeptidase (MAP) domain (Supplementary Figure S1). The *HbEBP1* gene sequence was then deposited into the GenBank (Accession No: KX661028). A phenogram generated by MEGA analysis demonstrated that EBP1 proteins are highly conserved in eukaryotic species and that EBP1s from plant and animal species are clustered respectively. Rubber tree EBP1 is highly similar to EBP1 from *Ricinus communis* (accession no. XP_002530504.1) and *Jatropha curcas* (accession no. XP_012089820.1), which also belong to the *Euphorbiaceae* family (**Figure 1B**).

### *HbEBP1* Expression Is Affected by Cold and Drought Stress and ABA Treatment

**FIGURE 2:**
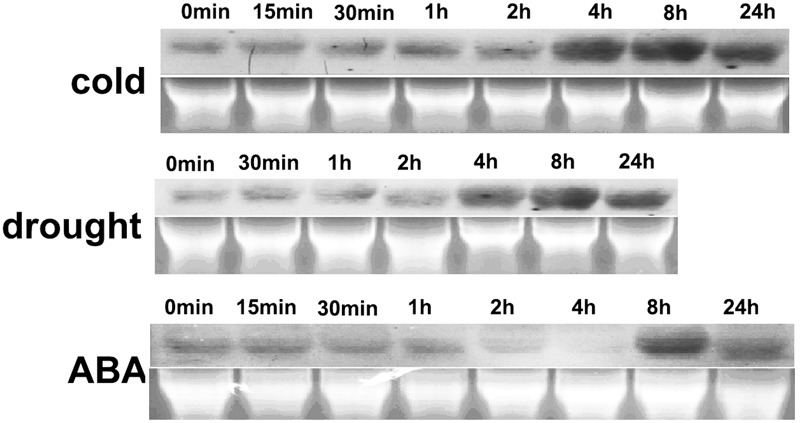
**Changes in *HbEBP1* expression in response to cold and drought stress and ABA treatment.** Total RNA from the leaves of rubber tree seedlings treated with cold, drought or ABA for the indicated time was used for northern blotting With probes prepared from full-length *HbEBP1* cDNA. Ethidium bromide–stained rRNA was used as an internal standard to monitor equal loading of total RNA.

The gene expression profiles under abiotic stress and ABA treatment were analyzed using northern blotting. *HbEBP1* expression was moderately expressed under ambient cultural conditions and this expression increased when rubber tree plants were subjected to cold, drought or ABA exposure. *HbEBP1* transcripts increased notably after 4 h of treatment with cold stress and reached their highest level at 8 h, with some decrease after 24 h of treatment (**Figure 2**). A similar expression pattern was found when the seedlings were subjected to drought stress, i.e., induction occurred after 4 h of treatment and reached its highest level after 8 h (**Figure 2**). During ABA treatment, *HbEBP1* expression showed some decrease during the first 4 h, followed by an increase after 8 h (**Figure 2**). *EBP1* genes accumulated in response to auxin in *Arabidopsis* and tomato, and their expression correlated with genes involved in ribosome biogenesis and function (Zhang et al., 2005; Horváth et al., 2006). The responses toward abiotic stress and ABA suggest that *HbEBP1* may be involved in the regulation of abiotic stress resistance in rubber trees.

### Overexpression of *HbEBP1* in *Arabidopsis* Leads to Enlarged Organ Size

**FIGURE 3:**
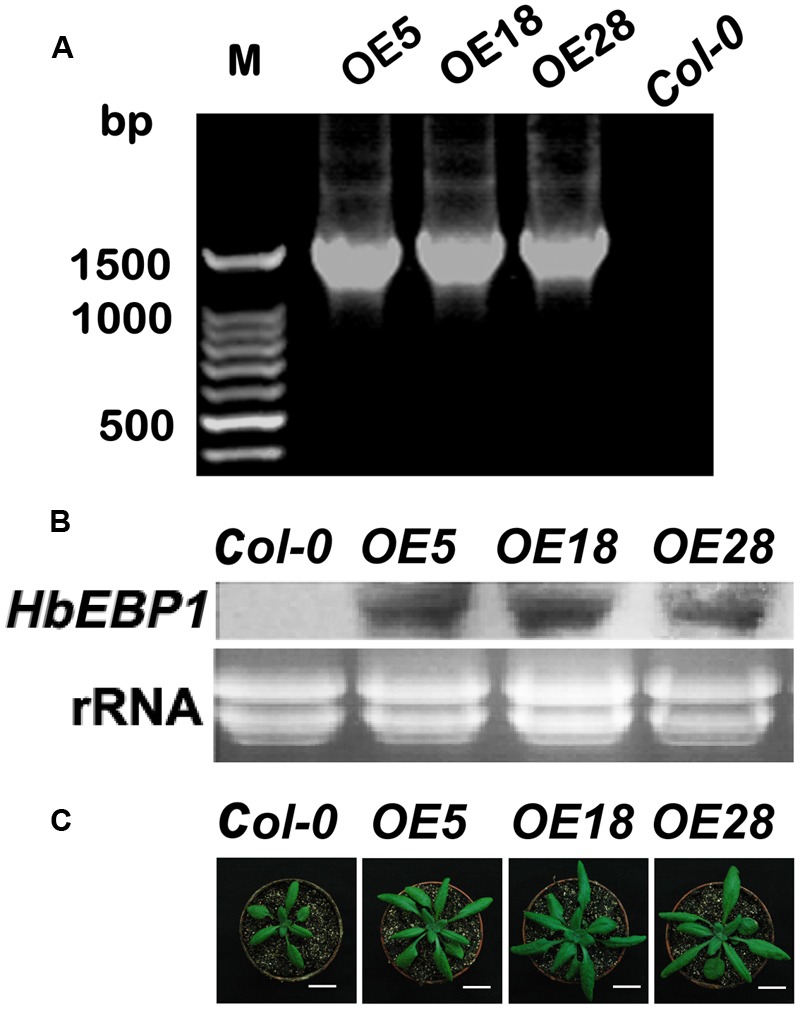
***HbEBP1* overexpression in *Arabidopsis*. (A)** PCR of *HbEBP1* in transgenic plants. **(B)** *HbEBP1* expression in the *OE5, OE18* and *OE28* lines. *HbEBP1* expression was detected by northern blotting analysis using *HbEBP1* probe. **(C)** Twenty-day-old seedlings from the *Col-0, OE5, OE18* and *OE28* lines showed enlarged rosette leaves. Bar = 2 cm.

The *EBP1* genes regulate organ size by stimulating both cell proliferation and expansion via the regulation of *RBR1* levels (Horváth et al., 2006). To validate these functions, *HbEBP1* was overexpressed in *Arabidopsis* using the floral dip method. PCR amplification was used to confirm gene transfer. Among tens of *HbEBP1* OE lines, we selected *OE5, OE18* and *OE28*, each of which contained a single-copy T-DNA insertion and showed high and stable expression, for further study (**Figure 3A**). The T3 generation seedlings that harbored homozygous T-DNA insertions were used for further analysis. Northern blotting was used to detect *HbEBP1* expression in the transgenic lines. As shown in **Figure 3B**, the *OE5, OE18* and *OE28* lines displayed comparable high expression. As expected, OE of *HbEBP1* led to markedly enlarged organ size in *Arabidopsis* seedlings. Leaves from all three OE lines were notably larger as compared with leaves from the *Col-0* seedlings (**Figure 3C**).

**FIGURE 4:**
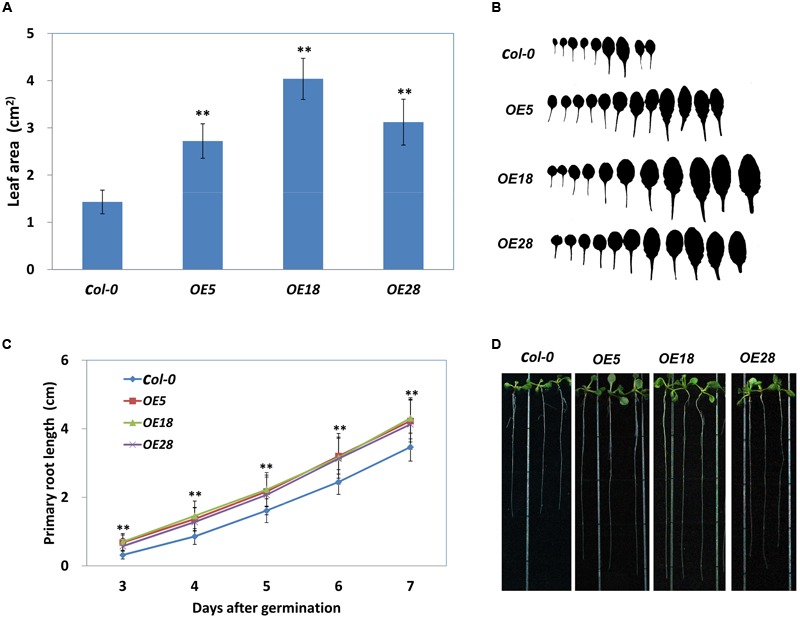
**Enlarged organ size in *Arabidopsis* plants that overexpress *HbEBP1*. (A)** Leaf area of the first adult-stage leaf (the seventh leaf) in the *Col-0, OE5, OE18* and OE28 lines. Leaves from at least 20 individual seedlings were measured for each line. **(B)** The morphology of rosette leaves from the *Col-0, OE5, OE18* and *OE28* seedlings arranged from left to right in the order of initiation. **(C)** Primary root elongation was faster in the *OE5, OE18* and *OE28* lines. The seedlings were cultured vertically, and the root length was measured daily. Roots from at least 20 individual seedlings were measured for each line. **(D)** Representative seedlings show the primary root length at 8 days after germination. Data in **(A,C)** were presented as mean ± standard error of mean of at least 20 individual seedlings. Student’s *t*-test was carried out for statistical analysis. Significant difference was defined by ^∗∗^*p* < 0.01.

The area of the first adult rosette leaf (normally the seventh leaf) was measured. As demonstrated in **Figures 4A,B**, the area of the seventh leaf was significantly larger in OE5, OE18 and OE28 lines, as compared with that in the *Col-0* plants (*p* < 0.01). The wild-type plants had an average leaf area of 1.43 ± 0.25 cm^2^, whereas the average leaf area from *OE5, OE18* and *OE28* plant was 2.72 ± 0.36, 4.04 ± 0.44 and 3.12 ± 0.49 cm^2^ respectively. In addition, the *HbEBP1* OE lines developed more rosette leaves than did the wild type *Col-0* seedlings (**Figure 4B**). We further measured the cell size and calculated total cell numbers. The results showed that the OE leaves have more cell numbers than the *Col-0*, while the cell size is comparable (Supplementary Figure S4). These results demonstrated *HbEBP1* OE promotes cell proliferation in transgenic lines.

Similar results were observed in the roots. Primary root length was measured daily, and roots were found to grow faster in *HbEBP1* transgenic seedlings. The *OE5, OE18* and *OE28* lines all displayed significantly faster root growth than did the *Col-0* seedlings from 3 days after germination (*p* < 0.01) (**Figure 4C**). By 8 days after germination, the *HbEBP1* OE lines had developed obviously longer primary roots than did the wild-type *Col-0* seedlings (**Figure 4D**). At 2 weeks of age, the root systems were more complex in the *HbEBP1* OE lines as compared with those in the *Col-0* plants (Supplementary Figure S2). Thus *HbEBP1* OE facilitated root development in *Arabidopsis*. The obviously larger leaves and longer primary roots in *HbEBP1* OE plants suggested that *HbEBP1* has a conserved function related to the regulation of organ size in *H. brasiliensis*.

### Overexpression of *HbEBP1* in *Arabidopsis* Leads to a Delayed Vegetative-to-Reproductive Transition and an Increased Adult Leaves

**FIGURE 5:**
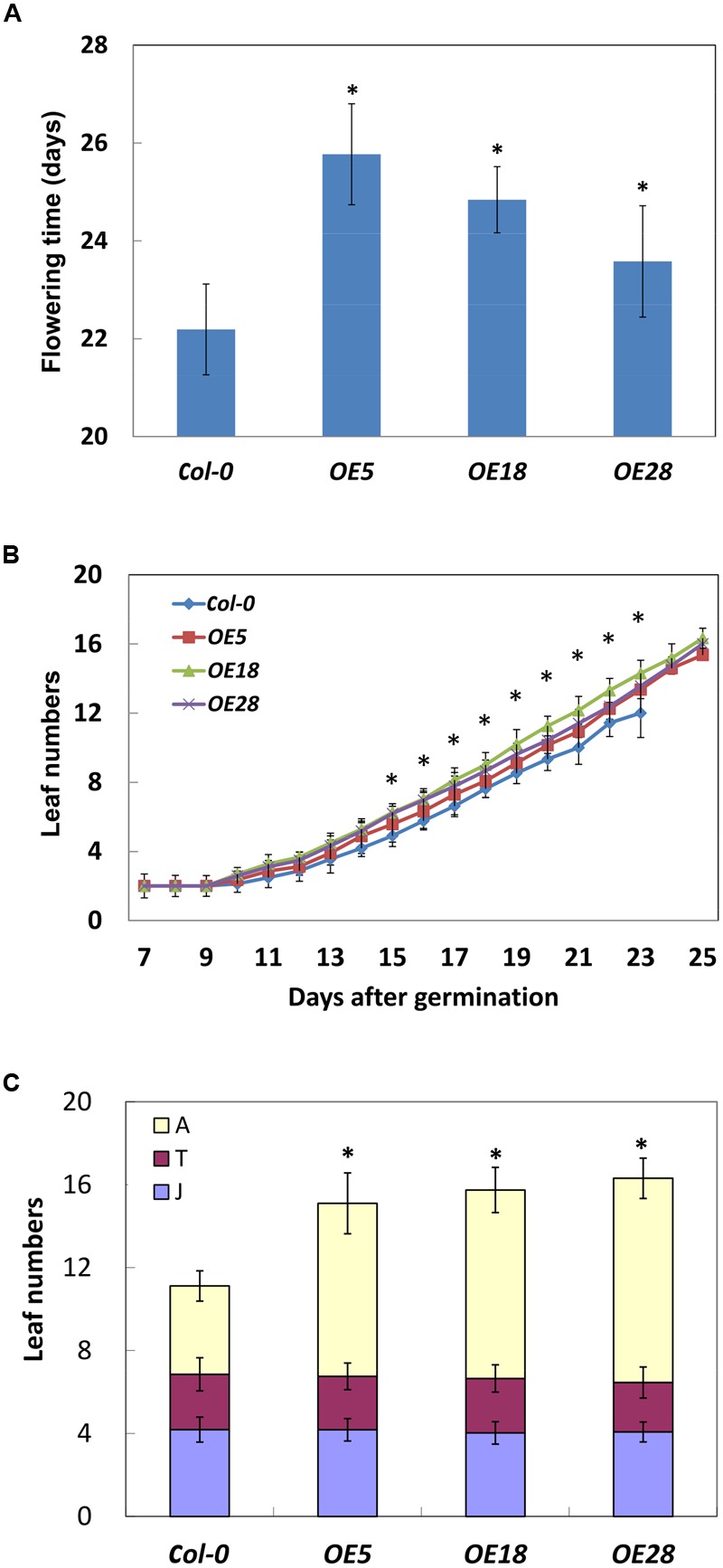
***HbEBP1* overexpression delayed the vegetative-to-reproductive transition in *Arabidopsis*. (A)** Days needed for the *Col-0, OE5, OE18* and *OE28* plants to produce visible flower meristems. **(B)** The leaf emergence rate is faster in the *OE5, OE18* and *OE28* lines than in *Col-0* plants. Leaf numbers were counted daily with naked eyes. **(C)** The number of rosette leaves in the phases of juvenile (J), juvenile-to-adult transition (T) and adult (A) leaves in the *Col-0, OE5, OE18* and *OE28* lines. Data were presented as mean ± SEM from at least twenty individual seedlings. Student’s *t*-test was carried out for statistical analysis. Significant difference was defined by ^∗^*p <* 0.05.

The *HbEBP1* OE lines displayed a late flowering phenotype. Under long-day conditions, the flower meristem was visible at 22.2 ± 0.9 days after planting for the *Col-0* plants. In contrast, for *OE5, OE18* and *OE28* lines, the flower meristem was visible at 25.8 ± 1.0, 24.8 ± 0.7 and 23.6 ± 1.1 days respectively. The flower meristems in the *HbEBP1 OE* lines were induced significantly later when compared with those from the wild-type plants (*p* < 0.05) (**Figure 5A**).

Late flowering prolonged the vegetative growth period in *HbEBP1* OE seedlings, which allowed the plants to grow more rosette leaves. An average of 15 or 16 leaves developed before floral induction for *HbEBP1* OE lines (25 days), whereas the number for the *Col-0* plants was 12 (22 days) (**Figure 5B**). We carefully counted rosette leaf number daily, and found that *HbEBP1* OE plants developed rosette leaves faster than did the *Col-0* plants. As shown in **Figure 5B**, all the *HbEBP1* OE lines grew significantly more leaves than *Col-0* from 15 days after germination (*p* < 0.01). Thus *HbEBP1* OE promoted leaf growth in *Arabidopsis*.

Late flowering is a result of prolonged vegetative growth, in which the juvenile-to-adult and vegetative-to-reproductive phase transitions are delayed (Martínez-Zapater et al., 1995; Poethig, 2003). To examine if the phase transitions were delayed in *HbEBP1* OE lines, leaf morphology was investigated at different phases. The leaf developmental stages were determined by observing the onset and the distribution of abaxial trichomes, leaf length and leaf shape (blade length/width ratio) (Telfer et al., 1997). The *OE5, OE18* and *OE28* lines had a comparable number of juvenile and juvenile-to-adult transition leaves relative to the *Col-0* plants (**Figure 5C**). Both the *HbEBP1* OE and wild-type plants developed about four juvenile and two juvenile-to-adult transition leaves. However, the number of leaves that developed during the adult phase was significantly higher for *OE5, OE18* and *OE28* plants (8.35 ± 1.47, 9.09 ± 1.09, and 9.85 ± 0.97, respectively; *p* < 0.01), whereas adult leaf number for the *Col-0* plants was 4.26 ± 0.73 (**Figure 5C**). Presumably the increase in the number of adult leaves and their enlarged size (**Figures 4A,B**) prolonged vegetative growth in the *HbEBP1* OE lines.

### Overexpression of *HbEBP1 in Arabidopsis* Enhances Resistance to Abiotic Stress

**FIGURE 6:**
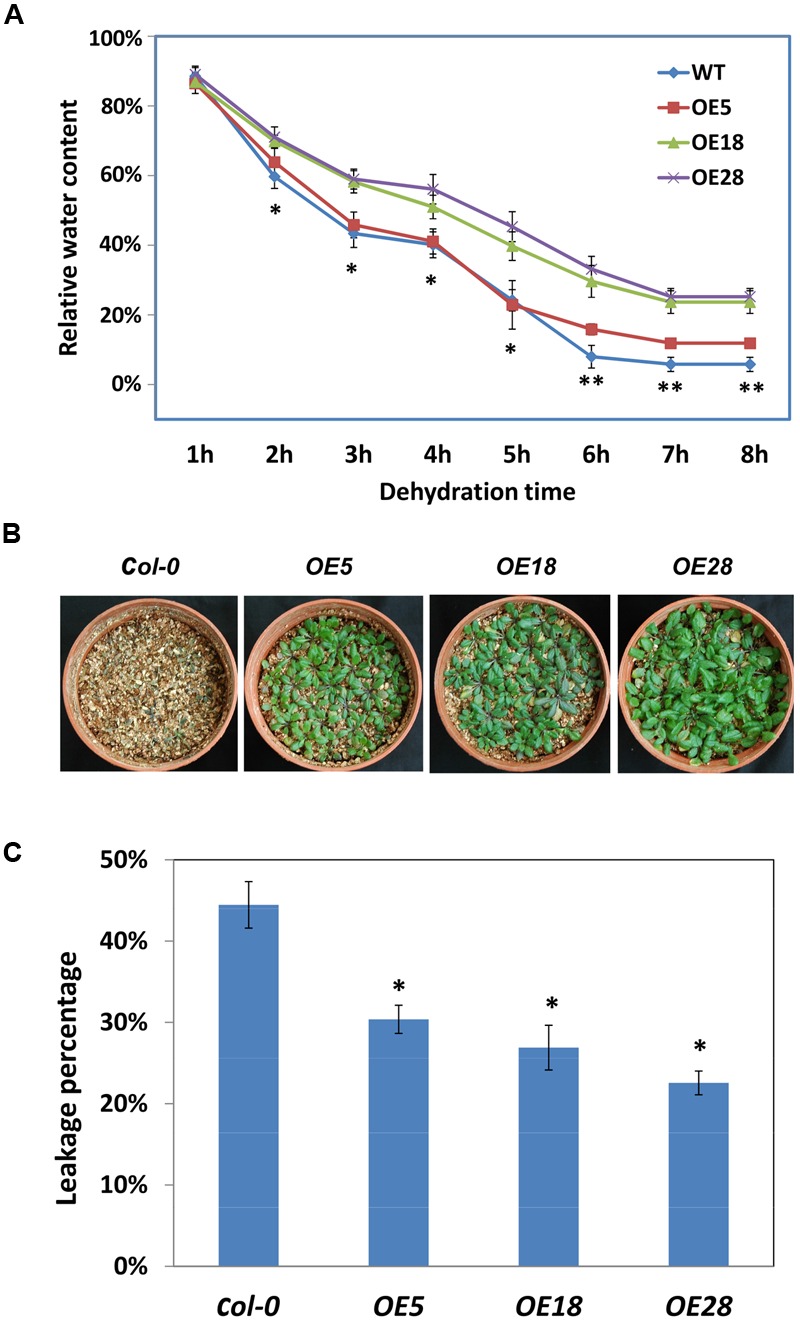
***HbEBP1* overexpression conferred drought and cold resistance in *Arabidopsis*. (A)** The dehydration rates of leaves from the *Col-0, OE5, OE18* and *OE28* seedlings. An asterisk (^∗^)indicates a significant difference between *Col-0* and the individual *OE18* and *OE28* lines, whereas a double asterisk (^∗∗^) indicates a significant difference between *Col-0* and each individual *HbEBP1* OE line. **(B)** Representative seedlings of the *Col-0, OE5, OE18* and *OE28* lines after irrigation were withheld for 7 days. The seedlings were grown in a pot containing vermiculite, and the drought stress treatment was conducted with 2-week-old seedlings by withdrawing water for 1 week. The photos were taken 3 days after re-irrigation. **(C)** Electrolyte leakage of leaves from the *Col-0, OE5, OE18* and *OE28* lines. Electrolyte leakage was measured using leaves from 2-week-old seedlings frozen to -7°C at a cooling rate of 1°C/h from -1°C. An asterisk (^∗^) indicates a significant difference between each individual *HbEBP1* OE line and the wild type *Col-0* seedlings. Data in **(A,C)** are presented as the mean ± SEM from five biological replicates. Significance was determined by the Student’s *t*-test at the probability levels of *p* < 0.05. Comparisons were made between *Col-0* and each individual OE line.

*HbEBP1* transcription was affected by cold and drought stress and ABA treatment, suggesting that this gene may be involved in abiotic resistance in plants. To assess its involvement in drought resistance, the dehydration rates were calculated for detached leaves. The *OE18* and *OE28* leaves dehydrated significantly slower than did the wild-type leaves from 2 to 8 h. For the *OE5* leaves, the dehydration rate was significantly slower from 6 to 8 h (**Figure 6A**). Thus *HbEBP1* OE plants might lose water more slowly than *Col-0* plants under drought conditions.

To evaluate drought tolerance *in vivo*, 2-week-old *HbEBP1* OE and *Col-0* seedlings were subjected to drought treatment by withdrawing water for 1 week. The *HbEBP1 OE5, OE18* and *OE28* lines showed a more resistant phenotype as compared with the wild-type plants (**Figure 6B**). After 7 days of withheld irrigation, all the *Col-0* plants were wilted and the rosette leaves had become chlorotic, whereas the *HbEBP1* OE lines had leaf blades that green and turgid, although their petioles were purple and displayed some aspects of a drought stressed phenotype (**Figure 6B**).

Similar results were obtained when *HbEBP1* OE plants were subjected to cold stress. Electrolyte leakage analysis was used to evaluate cold resistance. Significantly less leakage was detected in the *HbEBP1* OE plants when compared with the *Col-0* plants (*p* < 0.05). In this analysis, the control plants had a leakage rate of 44.5%, whereas the rates were 30.4, 26.9, and 22.6% for *OE5, OE18* and *OE28* lines, respectively. *HbEBP1* OE enhanced resistance to drought and cold stress in *Arabidopsis*, suggesting that this gene may function as a positive regulator of abiotic stress resistance in rubber trees.

### *HbEBP1* Overexpression Increased *CYCD3, RD22* and *RD29a* Transcripts in *Arabidopsis*

**FIGURE 7:**
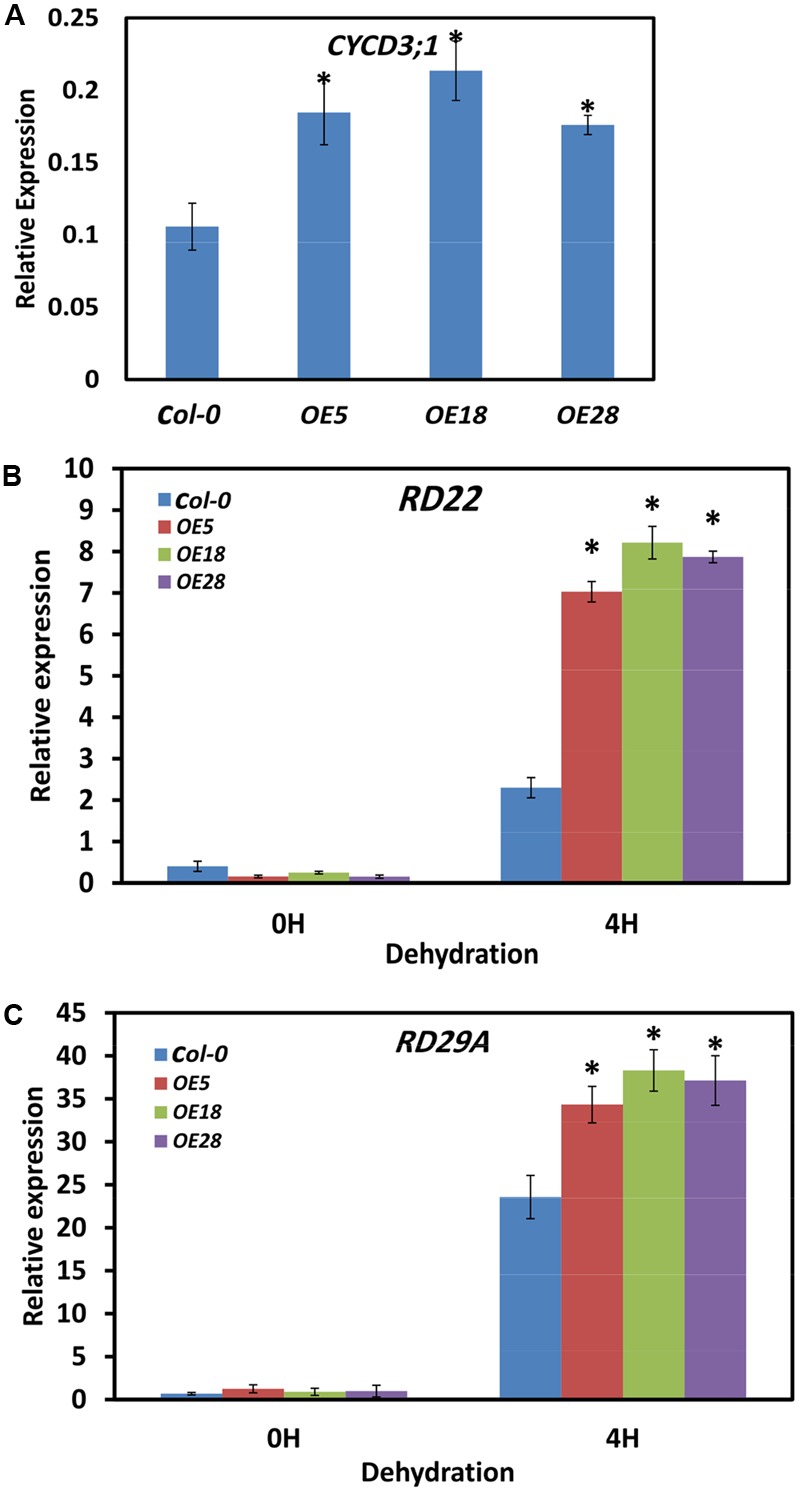
***HbEBP1* overexpression promoted the expression of *CYCD3;1, RD22* and *RD29a* in *Arabidopsis*. (A–C)** Quantification of expression of **(A)** *AtCYCD3;1*, **(B)** *AtRD22* and **(C)** *AtRD29a* by Q-PCR in the *Col-0, OE5, OE18* and *OE28* plants. For the dehydration treatment, the detached leaves were subjected to dehydration under the controlled condition for 4 h. Total RNA was extracted from 2-week-old seedling samples and Q-PCR was performed with gene-specific primers as listed in Supplementary Table S1. The transcript levels were normalized to that of *ACTIN7*. Two micrograms of total RNA was used for each sample analysis, and the data are presented as mean ± SEM from three biological replicates. An asterisk (^∗^) indicates a significant difference (*p* < 0.05) between the *HbEBP1* OE lines and *Col-0* in **(A)**, or between the *HbEBP1* OE lines and *Col-0* after 4 h of dehydration treatment in **(B,C)**. Significance was determined by Student’s *t*-test at the probability levels of *p* < 0.05.

*EBP1* regulates organ size by promoting cell cycle transitions during leaf development (Horváth et al., 2006). The expression of the critical cell cycle regulator of the G1 to S and G2 to M transitions, *CYCD3;1*, was examined by Q-PCR (Dewitte et al., 2007; Blomme et al., 2014; Collins et al., 2015). Two-week-old seedlings were used for the analysis, and gene expression was quantified by comparing the fold changes in expression between the genes of interest and the reference *ACTIN7*. In the *HbEBP1* OE lines, *CYCD3;1* expression were significantly increased by 1.7- to 2.1- fold as compared with that in the *Col-0* plants (**Figure 7A**). This effect may explain why *HbEBP1* OE led to enlarged organ size in *Arabidopsis*.

To unravel the mechanism by which *HbEBP1* OE enhances drought resistance in *Arabidopsis*, expression of the drought-responsive genes *RD22* and *RD29a* was examined (Yamaguchi-Shinozaki and Shinozaki, 1993b, 1994; Msanne et al., 2011; Harshavardhan et al., 2014). Leaves were detached from 2-week-old seedlings and subjected to dehydration for 4 h. Then the expression of dehydration-responsive genes was detected. In the seedlings without dehydration, *RD22* and *RD29a* expression was very low in both the *Col-0* and *HbEBP1* OE lines and showed no significant differences (*p* > 0.05; **Figures 7B,C**). After 4 h of dehydration, *RD22* and *RD29a* expression was highly induced in both the wild-type *Col-0* and *HbEBP1* OE lines, but the *HbEBP1* OE lines accumulated higher levels of transcripts than did the wild type plants. For *RD22*, the expression levels were 3.0-, 3.5- and 3.4- fold for *OE5, OE18* and *OE28* lines, respectively, when compared with that in the *Col-0* seedlings (**Figure 7B**). For *RD29a*, expression was increased by 45, 62, and 57% in *OE5, OE28* and *OE28* lines, respectively, relative to wild type (**Figure 7C**). The genes *CBF1, CBF2* and *CBF3*, which regulate *RD29a* (Yamaguchi-Shinozaki and Shinozaki, 1994; Stockinger et al., 1997; Jaglo-Ottosen et al., 1998; Liu et al., 1998; Medina et al., 1999), were also tested. Their expression did not differ between the wild-type and OE line seedlings (Supplementary Figure S3). These findings are similar to the reported effects of *AmEBP1* (Cao et al., 2008), demonstrating that the *HbEBP1* gene regulates drought resistance by other but not CBF pathway.

## Discussion

In plants, EBP1 regulates cell growth in a dose-dependent manner and requires the involvement of auxin (Horváth et al., 2006). The OE of *EBP1* often results in the enlargement of organs (Horváth et al., 2006; Wang et al., 2016). The OE of maize *EBP1* increases organ size by promoting cell proliferation in *Arabidopsis* (Wang et al., 2016). In this study, the *HbEBP1* OE lines develop leaves faster than the wild type seedlings (**Figure 5B**), and they have more cell number per leaf (Supplementary Figure S4), indicating that cell proliferation is accelerated in the *HbEBP1* OE plants. *HbEBP1* OE increased the expression of *CYCD3;1* in *Arabidopsis*, suggesting that *HbEBP1* has a conserved function in regulating the cell cycle. However, we also noticed that the *HbEBP1* OE lines exhibited a late flowering phenotype, which means that these plants had a longer vegetative growth phase. In plants, developmental progress includes distinct phases and a number of developmental transitions during their life cycle. The transitions between phases are controlled by several intrinsic and extrinsic cues (Amasino, 2010; Huijser and Schmid, 2011). When a plant passes through the juvenile-to-adult transition, it gains reproductive competency and gets ready for flowering (Poethig, 2003; Huijser and Schmid, 2011; Srikanth and Schmid, 2011). *HbEBP1* OE lines, with their late flowering phenotype, had more time to grow before passing through this transition. This is consistent with our observations concerning rosette leaf development in *HbEBP1* OE plants: the juvenile leaves did not differ from those in the wild-type seedlings, whereas the adult leaves had an accelerated rate of emergence, were present in higher numbers and had larger leaf blades (**Figures 4** and **5**). As a result, the accelerated cell proliferation and longer vegetative growth period increased both the leaf number and size.

*Ammopiptanthus mongolicus EBP1* is induced by cold, and confers enhanced cold tolerance when expressed in *E. coli*, as well as notably increased freezing survival when expressed in *Arabidopsis* (Cao et al., 2008). In this study, the rubber tree *EBP1* was also induced by cold, drought and ABA treatment (**Figure 2**), OE of *HbEBP1* conferred increased drought and freezing tolerance in *Arabidopsis* (**Figure 6**). Phenotypic characterization indicated that the *HbEBP1* OE lines had relatively slower dehydration rates as compared with wild-type seedlings. In addition, the OE lines had better-developed root systems (**Figure 4D**, Supplementary Figure S2). These developmental features may also contribute to the robust drought resistance in the *HbEBP1* OE lines.

Gene expression analysis indicated that *RD29a* and *RD22* expression was enhanced in the *HbEBP1* OE lines. *RD29a* and *RD22* are involved in drought stress in plants (Yamaguchi-Shinozaki and Shinozaki, 1993a,b; Thomashow, 1999). The up-regulation of *RD29a* and *RD22* thus suggested that *HbEBP1* gene is involved in the regulation of drought resistance. We also analyzed the expression of *CBF1–3*, regulators of *RD29a* (Yamaguchi-Shinozaki and Shinozaki, 1994; Stockinger et al., 1997; Jaglo-Ottosen et al., 1998; Liu et al., 1998; Medina et al., 1999), The absence of an effect on their expression in the *HbEBP1* OE lines (Supplementary Figure S3) is consistent with the effects of *AmEBP1* (Cao et al., 2008), demonstrating that *HbEBP1* regulates drought resistance by a mechanism that does not involve the CBF pathway.

Although the detailed regulatory pathway is still unknown, there has been speculation that EBP1 functions as a MAP and forms complexes with small molecules to accelerate protein processing after translation, which is essential for the plant to respond to abiotic stress (Cao et al., 2008). However, HbEBP1 when expressed in *E. coli* did not exhibit MAP activity *in vitro* (Cheng et al., 2016). This is consistent with the EBP1 crystal structure, which has the conserved pita bread fold of MAPs, although the protein lacks the characteristic enzymatic activity (Kowalinski et al., 2007; Monie et al., 2007). Therefore, even though EBP1 binds with dsRNA to form part of RNP complexes via association with different rRNA species in human cells (Squatrito et al., 2004), HbEBP1 may not possess the MAP activity needed to accelerate protein processing, which involves cutting off the first methionine from the peptide after translation in eukaryotic organisms (Datta, 2000). As EBP1 interacts with a number of proteins and RNAs that are involved in either transcription regulation or translation control (Squatrito et al., 2004, 2006; Ahn et al., 2006; Zhang et al., 2015), the MAP domain in HbEBP1 is more likely to function as a protein- or RNA-interacting motif (Monie et al., 2007).

In summary, the expression of an *EBP1* from tropical woody plants was shown to be induced by drought and cold stress treatment. *HbEBP1* was also able to promote drought and cold resistance, in addition to growth and organ size, in transgenic *Arabidopsis* plants. *HbEBP1* OE also increased the expression of drought resistance–related *RD22* and *RD29a* and of the cell cycle *CYCD3;1*. The regulation of drought resistance is a novel function identified in plant *EBP1* genes. Although the detailed regulatory mechanism has yet to be determined, *HbEBP1* may be useful in genetically engineered crops to increase both organ size (biomass) and abiotic stress resistance at the same time.

## Conclusion

The study reported here, for the first time, describes the identification and characterization of an *ErbB-3 binding protein 1* gene from *H. brasiliensis*. *HbEBP1* is conserved at the level of its amino acid sequence and protein domains with other reported *EBP1s*. When subjected to abiotic stress or ABA treatment, *HbEBP1* expression was induced, suggesting its roles in cold and drought resistance. Further transgenic experiment indicated that *HbEBP1* has conserved functions in enlarging organ size. In addition, *HbEBP1* OE delayed flowering time and the vegetative-to-reproductive transition and increased the adult leaf number. More importantly, the *HbEBP1* OE lines showed enhanced resistance to freezing and drought stress, which is a novel function identified in plant *EBP1* genes. Further analysis revealed that *CYCD3;1, RD29a* and *RD22* expression was promoted in the *HbEBP1* OE lines, which helps to explain the regulatory roles of *HbEBP1*. Taking together, *HbEBP1* may be useful in genetically engineered crops to increase both organ size (biomass) and abiotic stresses resistance at the same time.

## Author Contributions

HC and HH designed the experiments; JZ and XC conducted the experiments; HC wrote the manuscript draft; HH discussed the results and finalized the manuscript.

## Conflict of Interest Statement

The authors declare that the research was conducted in the absence of any commercial or financial relationships that could be construed as a potential conflict of interest.

## Footnotes

1 http://www.ebi.ac.uk/Tools/msa/clustalw2/

2 http://blast.ncbi.nlm.nih.gov/Blast.cgi

3 http://www.ebi.ac.uk/Tools/pfa/iprscan5/

## Abbreviations

ABA: abscisic acid
EBP1: ErbB-3 binding protein 1
OE: overexpression

## References

[B1] Ahn J.-Y. Liu X. Liu Z. Pereira L. Cheng D. Peng J. (2006). Nuclear Akt associates with PKC-phosphorylated Ebp1, preventing DNA fragmentation by inhibition of caspase-activated DNase. *EMBO J.* 25 2083–2095. 10.1038/sj.emboj.7601111 16642037 PMC1462972

[B2] Amasino R. (2010). Seasonal and developmental timing of flowering. *Plant J.* 61 1001–1013. 10.1111/j.1365-313X.2010.04148.x 20409274

[B3] Blomme J. Inzé D. Gonzalez N. (2014). The cell-cycle interactome: a source of growth regulators? *J. Exp. Bot.* 65 2715–2730. 10.1093/jxb/ert388 24298000

[B4] Cao P. Song J. Zhou C. Weng M. Liu J. Wang F. (2008). Characterization of multiple cold induced genes from *Ammopiptanthus mongolicus* and functional analyses of gene AmEBP1. *Plant Mol. Biol.* 69 529–539. 10.1007/s11103-008-9434-1 19067182

[B5] Cheng H. Cai H. An Z. Huang H. (2013). Comparative proteomics analysis revealed increased expression of photosynthetic proteins in transgenic tobacco by overexpression of AtCBF1 gene. *Plant Omics* 6 240–245.

[B6] Cheng H. Cai H. Fu H. An Z. Fang J. Hu Y. (2015). Functional characterization of *Hevea brasiliensis* CRT/DRE binding factor 1 gene revealed regulation potential in the CBF pathway of tropical perennial tree. *PLoS ONE* 10:e0137634 10.1371/journal.pone.0137634 PMC4567348 26361044

[B7] Cheng H. Cai H. Huang H. (2008). Construction of full-length cDNA library in rubber tree under cold stress. *Chin. J. Trop. Crops* 41 410–414.

[B8] Cheng H. Zhu J. An Z. Fang J. Hu Y. Huang H. (2016). The characterization, prokaryotic expression and MetAP activity analysis of *Hevea brasiliensis* HbEBP1. *Chin. J. Trop. Crops* 37 317–324.

[B9] Clough S. J. Bent A. F. (1998). Floral dip: a simplified method for *Agrobacterium*-mediated transformation of *Arabidopsis thaliana*. *Plant J.* 16 735–743. 10.1046/j.1365-313x.1998.00343.x 10069079

[B10] Collins C. Maruthi N. M. Jahn C. E. (2015). CYCD3 D-type cyclins regulate cambial cell proliferation and secondary growth in *Arabidopsis*. *J. Exp. Bot.* 66 4595–4606. 10.1093/jxb/erv218 26022252 PMC4507761

[B11] Datta B. (2000). MAPs and POEP of the roads from prokaryotic to eukaryotic kingdoms. *Biochimie* 82 95–107. 10.1016/S0300-9084(00)00383-7 10727764

[B12] Dewitte W. Scofield S. Alcasabas A. A. Maughan S. C. Menges M. Braun N. (2007). *Arabidopsis* CYCD3 D-type cyclins link cell proliferation and endocycles and are rate-limiting for cytokinin responses. *Proc. Natl. Acad. Sci. U.S.A.* 104 14537–14542. 10.1073/pnas.0704166104 17726100 PMC1964848

[B13] Figeac N. Serralbo O. Marcelle C. Zammit P. S. (2014). ErbB3 binding protein-1 (Ebp1) controls proliferation and myogenic differentiation of muscle stem cells. *Dev. Biol.* 386 135–151. 10.1016/j.ydbio.2013.11.017 24275324

[B14] Harshavardhan V. T. Van Son L. Seiler C. Junker A. Weigelt-Fischer K. Klukas C. (2014). AtRD22 and AtUSPL1, members of the plant-specific BURP domain family involved in *Arabidopsis thaliana* drought tolerance. *PLoS ONE* 9:e110065 10.1371/journal.pone.0110065 PMC4198191 25333723

[B15] Horváth B. M. Magyar Z. Zhang Y. Hamburger A. W. Bakó L. Visser R. G. F. (2006). EBP1 regulates organ size through cell growth and proliferation in plants. *EMBO J.* 25 4909–4920. 10.1038/sj.emboj.7601362 17024182 PMC1618091

[B16] Huijser P. Schmid M. (2011). The control of developmental phase transitions in plants. *Development* 138 4117–4129. 10.1242/dev.063511 21896627

[B17] Jaglo-Ottosen K. R. Gilmour S. J. Zarka D. G. Schabenberger O. Thomashow M. F. (1998). *Arabidopsis* CBF1 overexpression induces COR genes and enhances freezing tolerance. *Science* 280 104–106. 10.1126/science.280.5360.104 9525853

[B18] Jones P. Binns D. Chang H.-Y. Fraser M. Li W. McAnulla C. (2014). InterProScan 5: genome-scale protein function classification. *Bioinformatics* 30 1236–1240. 10.1093/bioinformatics/btu031 24451626 PMC3998142

[B19] Kowalinski E. Bange G. Bradatsch B. Hurt E. Wild K. Sinning I. (2007). The crystal structure of Ebp1 reveals a methionine aminopeptidase fold as binding platform for multiple interactions. *FEBS Lett.* 581 4450–4454. 10.1016/j.febslet.2007.08.024 17765895

[B20] Lamartine J. Seri M. Cinti R. Heitzmann F. Creaven M. Radomski N. (1997). Molecular cloning and mapping of a human cDNA (PA2G4) that encodes a protein highly homologous to the mouse cell cycle protein p38-2G4. *Cytogenet. Cell Genet.* 78 31–35. 10.1159/000134621 9345902

[B21] Larkin M. A. Blackshields G. Brown N. P. Chenna R. McGettigan P. A. McWilliam H. (2007). Clustal W and Clustal X version 2.0. *Bioinformatics* 23 2947–2948. 10.1093/bioinformatics/btm404 17846036

[B22] Lessor T. J. Yoo J.-Y. Xia X. Woodford N. Hamburger A. W. (2000). Ectopic expression of the ErbB-3 binding protein Ebp1 inhibits growth and induces differentiation of human breast cancer cell lines. *J. Cell. Physiol.* 183 321–329. 10.1002/(SICI)1097-4652(200006)183:3<321::AID-JCP4>3.0.CO;2-O 10797306

[B23] Liu Q. Kasuga M. Sakuma Y. Abe H. Miura S. Yamaguchi-Shinozaki K. (1998). Two transcription factors, DREB1 and DREB2, with an EREBP/AP2 DNA binding domain separate two cellular signal transduction pathways in drought- and low-temperature-responsive gene expression, respectively, in *Arabidopsis*. *Plant Cell* 10 1391–1406. 10.1105/tpc.10.8.1391 9707537 PMC144379

[B24] Liu Y. Liu Y. Cao J. Zhu X. Nie X. Yao L. (2014). Upregulated expression of Ebp1 contributes to schwann cell differentiation and migration after sciatic nerve crush. *J. Mol. Neurosci.* 54 602–613. 10.1007/s12031-014-0331-6 24878627

[B25] Liu Z. Ahn J.-Y. Liu X. Ye K. (2006). Ebp1 isoforms distinctively regulate cell survival and differentiation. *Proc. Natl. Acad. Sci. U.S.A.* 103 10917–10922. 10.1073/pnas.0602923103 16832058 PMC1544149

[B26] Martínez-Zapater J. M. Jarillo J. A. Cruz-Alvarez M. Roldán M. Salinas J. (1995). *Arabidopsis* late-flowering fve mutants are affected in both vegetative and reproductive development. *Plant J.* 7 543–551. 10.1046/j.1365-313X.1995.7040543.x

[B27] Medina J. Bargues M. Terol J. Perez-Alonso M. Salinas J. (1999). The *Arabidopsis* CBF gene family is composed of three genes encoding AP2 domain-containing proteins whose expression is regulated by low temperature but not by abscisic acid or dehydration. *Plant Physiol.* 119 463–470. 10.1104/pp.119.2.463 9952441 PMC32122

[B28] Monie T. P. Perrin A. J. Birtley J. R. Sweeney T. R. Karakasiliotis I. Chaudhry Y. (2007). Structural insights into the transcriptional and translational roles of Ebp1. *EMBO J.* 26 3936–3944. 10.1038/sj.emboj.7601817 17690690 PMC1994118

[B29] Montoro P. Rattana W. Pujade-Renaud V. Michaux-Ferrière N. Monkolsook Y. Kanthapura R. (2003). Production of *Hevea brasiliensis* transgenic embryogenic callus lines by *Agrobacterium tumefaciens*: roles of calcium. *Plant Cell Rep.* 21 1095–1102. 10.1007/s00299-003-0632-7 12836004

[B30] Msanne J. Lin J. Stone J. M. Awada T. (2011). Characterization of abiotic stress-responsive *Arabidopsis thaliana* RD29A and RD29B genes and evaluation of transgenes. *Planta* 234 97–107. 10.1007/s00425-011-1387-y 21374086

[B31] Nguyen L. X. T. Lee Y. Urbani L. Utz P. J. Hamburger A. W. Sunwoo J. B. (2015). Regulation of ribosomal RNA synthesis in T cells: requirement for GTP and Ebp1. *Blood* 125 2519–2529. 10.1182/blood-2014-12-616433 25691158 PMC4400289

[B32] Poethig R. S. (2003). Phase change and the regulation of developmental timing in plants. *Science* 301 334–336. 10.1126/science.1085328 12869752

[B33] Schneider C. A. Rasband W. S. Eliceiri K. W. (2012). NIH Image to ImageJ: 25 years of image analysis. *Nat. Methods* 9 671–675. 10.1038/nmeth.2089 22930834 PMC5554542

[B34] Squatrito M. Mancino M. Donzelli M. Areces L. B. Draetta G. F. (2004). EBP1 is a nucleolar growth-regulating protein that is part of pre-ribosomal ribonucleoprotein complexes. *Oncogene* 23 4454–4465. 10.1038/sj.onc.1207579 15064750

[B35] Squatrito M. Mancino M. Sala L. Draetta G. F. (2006). Ebp1 is a dsRNA-binding protein associated with ribosomes that modulates eIF2α phosphorylation. *Biochem. Biophys. Res. Commun.* 344 859–868. 10.1016/j.bbrc.2006.03.205 16631606

[B36] Srikanth A. Schmid M. (2011). Regulation of flowering time: all roads lead to Rome. *Cell. Mol. Life Sci.* 68 2013–2037. 10.1007/s00018-011-0673-y 21611891 PMC11115107

[B37] Stockinger E. J. Gilmour S. J. Thomashow M. F. (1997). *Arabidopsis thaliana* CBF1 encodes an AP2 domain-containing transcriptional activator that binds to the C-repeat/DRE, a cis-acting DNA regulatory element that stimulates transcription in response to low temperature and water deficit. *Proc. Natl. Acad. Sci. U. S. A.* 94 1035–1040. 10.1073/pnas.94.3.1035 9023378 PMC19635

[B38] Sun J. Luo Y. Tian Z. Gu L. Xia S. C. Yu Y. (2012). Expression of ERBB3 binding protein 1 (EBP1) in salivary adenoid cystic carcinoma and its clinicopathological relevance. *BMC Cancer* 12:499 10.1186/1471-2407-12-499 PMC3499390 23110497

[B39] Tamura K. Stecher G. Peterson D. Filipski A. Kumar S. (2013). MEGA6: molecular evolutionary genetics analysis version 6.0. *Mol. Biol. Evol.* 30 2725–2729. 10.1093/molbev/mst197 24132122 PMC3840312

[B40] Tang C. Yang M. Fang Y. Luo Y. Gao S. Xiao X. (2016). The rubber tree genome reveals new insights into rubber production and species adaptation. *Nat. Plants* 2 16073 10.1038/nplants.2016.73 27255837

[B41] Telfer A. Bollman K. M. Poethig R. S. (1997). Phase change and the regulation of trichome distribution in *Arabidopsis thaliana*. *Development* 124 645–654. 9043079 10.1242/dev.124.3.645

[B42] Thomashow M. F. (1999). PLANT COLD ACCLIMATION: freezing tolerance genes and regulatory mechanisms. *Annu. Rev. Plant Physiol. Plant Mol. Biol.* 50 571–599. 10.1146/annurev.arplant.50.1.571 15012220

[B43] Wang T. Sui Z. Liu X. Li Y. Li H. Xing J. (2016). Ectopic expression of a maize hybrid up-regulated gene, ErbB-3 binding Protein 1 (ZmEBP1), increases organ size by promoting cell proliferation in *Arabidopsis*. *Plant Sci.* 243 23–34. 10.1016/j.plantsci.2015.11.002 26795148

[B44] Willmann M. R. Poethig R. S. (2011). The effect of the floral repressor FLC on the timing and progression of vegetative phase change in *Arabidopsis*. *Development* 138 677–685. 10.1242/dev.057448 21228003 PMC3026413

[B45] Xia X. Lessor T. J. Zhang Y. Woodford N. Hamburger A. W. (2001). Analysis of the expression pattern of Ebp1, an ErbB-3-binding protein. *Biochem. Biophys. Res. Commun.* 289 240–244. 10.1006/bbrc.2001.5942 11708806

[B46] Yamaguchi-Shinozaki K. Shinozaki K. (1993a). *Arabidopsis* DNA encoding two desiccation-responsive rd29 genes. *Plant Physiol.* 101 1119–1120. 10.1104/pp.101.3.1119 8310052 PMC158736

[B47] Yamaguchi-Shinozaki K. Shinozaki K. (1993b). The plant hormone abscisic acid mediates the drought-induced expression but not the seed-specific expression of rd22, a gene responsive to dehydration stress in *Arabidopsis thaliana*. *Mol. Gen. Genet.* 238 17–25. 8479424 10.1007/BF00279525

[B48] Yamaguchi-Shinozaki K. Shinozaki K. (1994). A novel cis-acting element in an *Arabidopsis* gene is involved in responsiveness to drought, low-temperature, or high-salt stress. *Plant Cell* 6 251–264. 10.1105/tpc.6.2.251 8148648 PMC160431

[B49] Zewei An (2012). Co-extraction of high-quality RNA and DNA from rubber tree (*Hevea brasiliensis*). *Afr. J. Biotechnol.* 11 9308–9314. 10.5897/AJB12.136

[B50] Zhang F. Liu Y. Wang Z. Sun X. Yuan J. Wang T. (2015). A novel Anxa2-interacting protein Ebp1 inhibits cancer proliferation and invasion by suppressing Anxa2 protein level. *Mol. Cell. Endocrinol.* 411 75–85. 10.1016/j.mce.2015.04.013 25917452

[B51] Zhang W.-K. Shen Y.-G. He X.-J. Du B.-X. Xie Z.-M. Luo G.-Z. (2005). Characterization of a novel cell cycle-related gene from *Arabidopsis*. *J. Exp. Bot.* 56 807–816. 10.1093/jxb/eri075 15689342

