# Supplementary material for: Overexpression of a Hevea brasiliensis ErbB-3 Binding protein 1 Gene Increases Drought Tolerance and Organ Size in Arabidopsis
Source: Front Plant Sci. 2016 Nov 14;7:1703. doi: 10.3389/fpls.2016.01703 (PMC5107689; doi:10.3389/fpls.2016.01703)
Supplement: Supplementary file 1 [file Data_Sheet_1.doc]

Supplementary Material

# Overexpression of a *Hevea brasiliensis* ErbB-3 Binding Protein 1 Gene Increases Drought Tolerance and Enlarges Organ Size in *Arabidopsis*

Cheng Han, Chen Xiang, Zhu Jianshun, Huang Huasun

Supplementary Tables

**1.1 Supplemental** Table S1. Oligo used in this study

| Primer | Sequence (5′–3′) |
| --- | --- |
| HbEBP1-CDS-F | TCAAAGCTGTAAGCTTATGTCGG |
| HbEBP1-CDS-R | CATAAGAATTCCATACAAGGT |
| HbEBP1-F | GGCTAGCAGTCCAGTGAGCCAC |
| HbEBP1-R | CTATCAATATGAAGGGAAATTCA |
| AtRD29A-F | TTGCCGAGAAACTTCAGATTG |
| AtRD29A-A | ACCACCGAACCATCCTTTA |
| AtCOR15A-F | ATCTACGCCGCTAAAGG |
| AtCOR15A-A | AATGTATCTGCGGTTTCACT |
| AtCBF1-f | GTGATACGACGACCACG |
| AtCBF1-A | AACAAAGTCGGCATCCC |
| AtCBF2-F | GAGGCTATTTATACGCCGGA |
| AtCBF2-A | CATAAGGACACGTCATCATCTC |
| AtCBF3-F | GCTATTTACACGGCGGAAC |
| AtCBF3-A | CCATAACGATACGTCGTCA |
| AtRD22-F | CAAGAGCATTTAAGGAGCAGAAGGCACG |
| AtRD22-R | CCAAGAAGACAGATCAGAGGAAGCCGAA |
| ACTIN7-F | GTCCCTGCCATGTATGTT |
| ACTIN7-A | CCCGCAAGATCAAGACG |
| AtCYCD3;1-F | CAATGCTCACTGGGATTTCCTCAAC |
| AtCYCD3;1-R | GGTATGAAAGAGGGTCAAAGGGAT |
| Lib-3 | TGTAGAGAGAGACTGGTGATTTTTG |
| Lib-5 | ATGACGCACAATCCCACTATC |

# Supplemental Figures


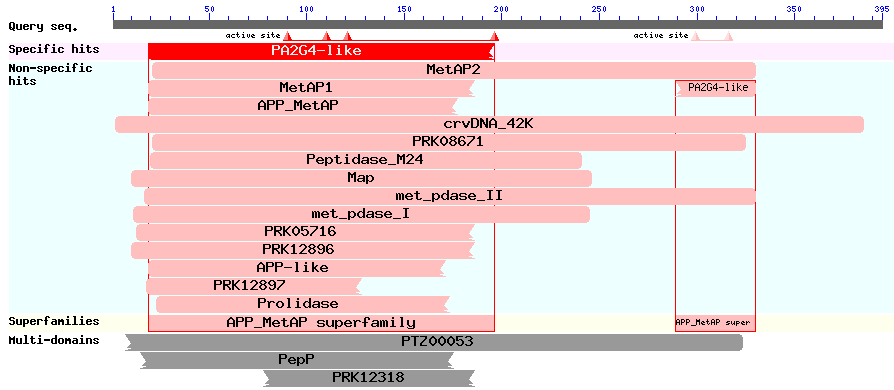


**2.1 Supplemental Figure 1. Proposed domains in HbEBP1 protein.**


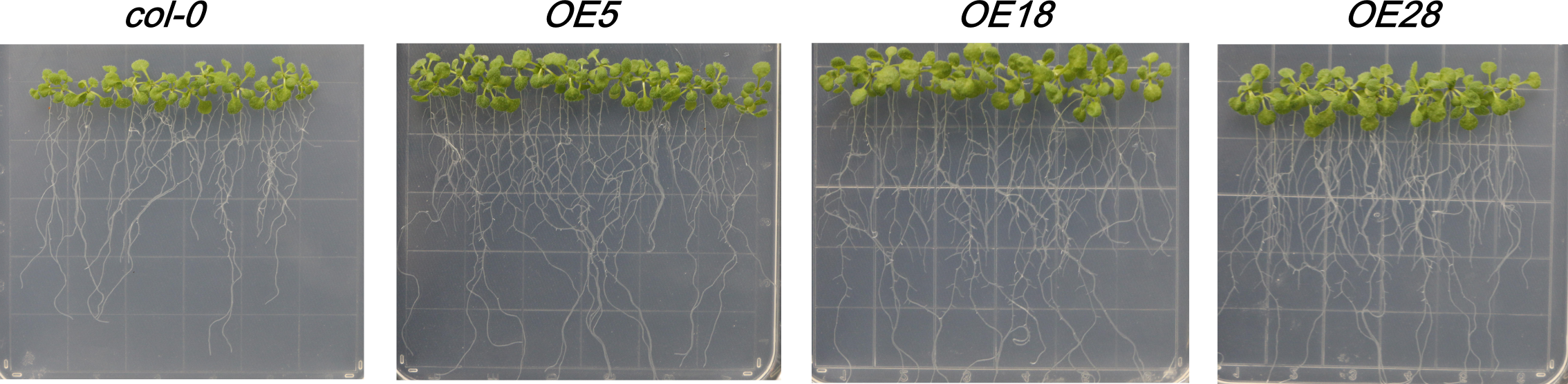


**2.2 Supplemental Figure 2.** **The root system of *Col-0* and *HbEBP1* OE lines.** The seeds were sowed onto the square plates and incubated in at 4 °C for 4 days, then were cultured vertically. The photos were taken after 12 days in culture. The root systems were apparently more well-developed in OE lines than in wild type *Col-0* seedlings.


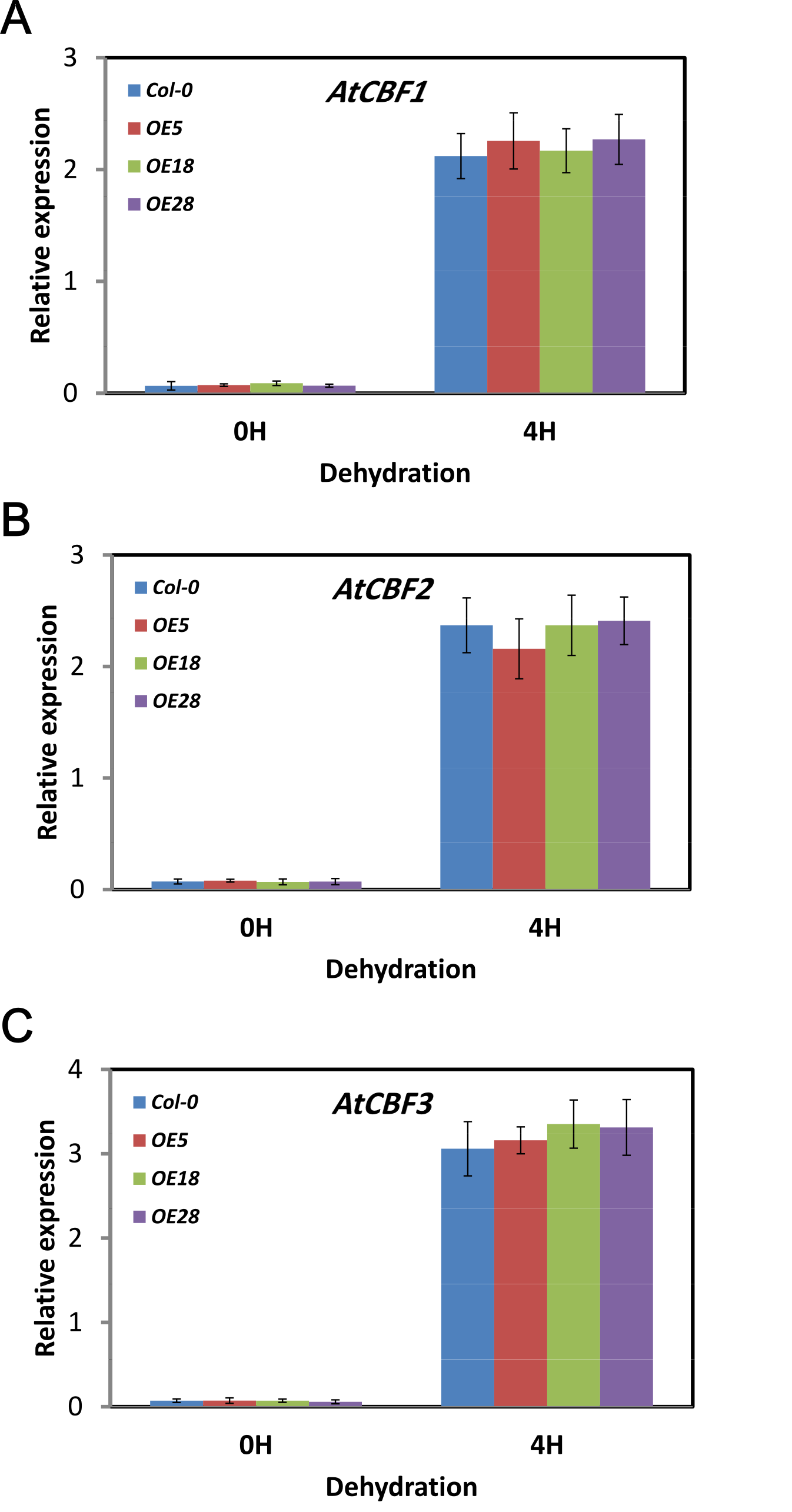


**2.3 Supplemental Figure 3. *HbEBP1* overexpression did not affect the expression of *CBF1, CBF2 and CBF3* in *Arabidopsis*.** (A-C) Expression of (A) *AtCBF1*, (B) *AtCBF2* and (C) *AtCBF3* expression in the *Col-0*, *OE5*, *OE18* and *OE28* plants. Data were generated and analyzed as described for Figure 7; primer sequences are in Supplemental Table 1. Significance was determined by Student's *t* test at the probability levels of *p* < 0.05.

**
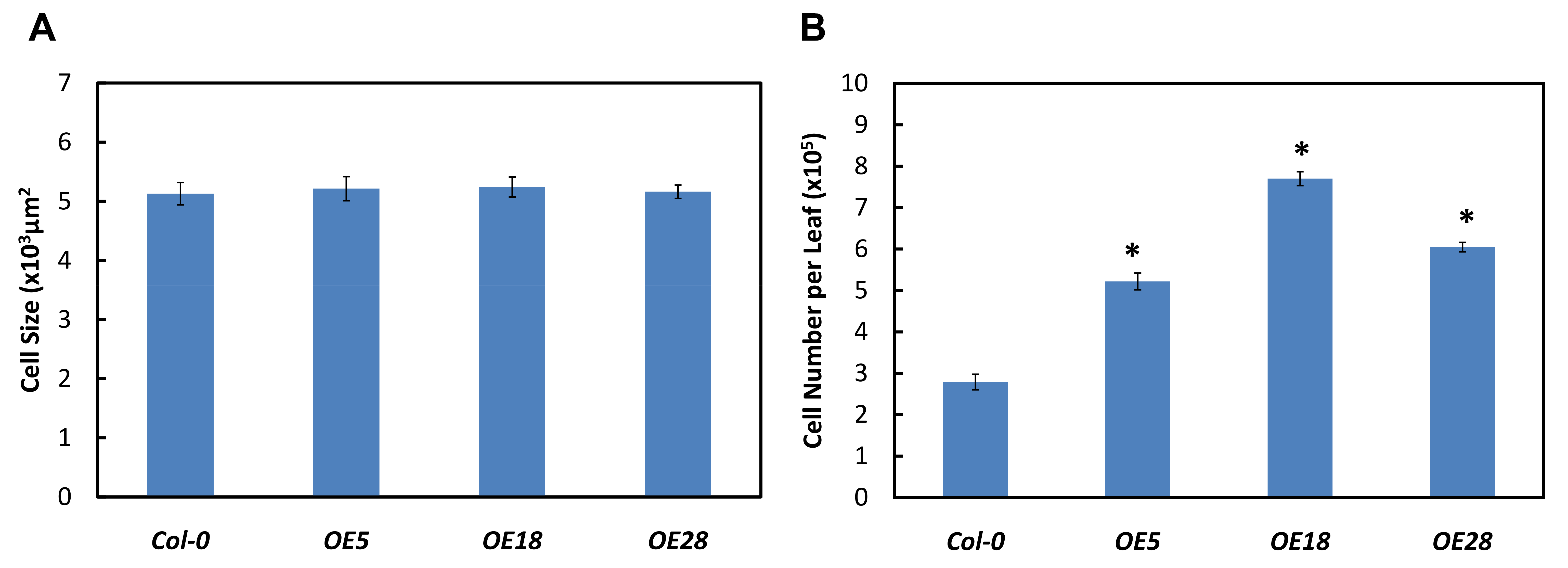
**

**
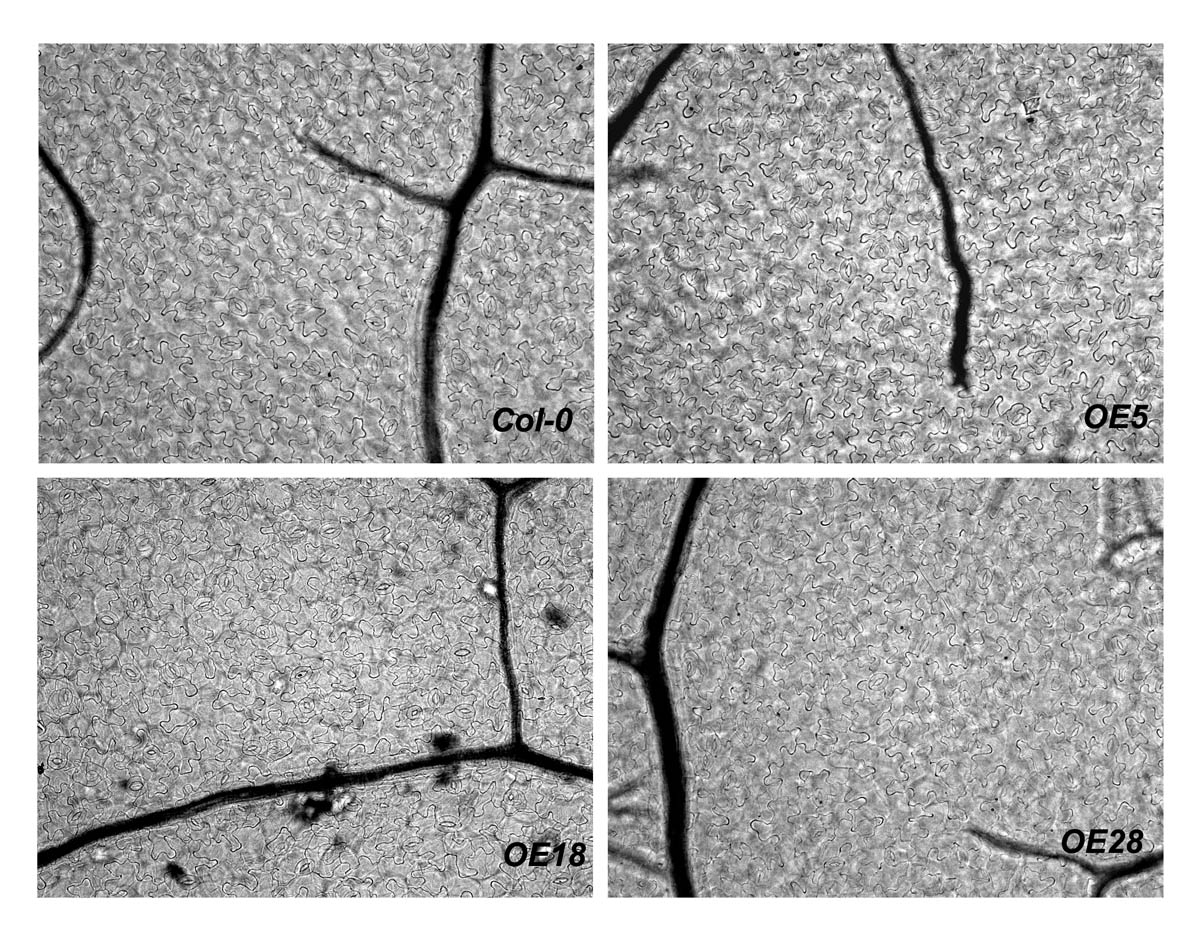
**

**C**

**2.4 Supplemental Figure 4. *HbEBP1* overexpression promoted cell proliferation in *Arabidopsis*.** (A) Total cell numbers and (B) epidermal cell size in *HbEBP1* OE lines and wild-type *Arabidopsis* seedlings. Ten *Arabidopsis* plants were selected randomly. The area of the fully expanded seventh leaf was measured at about 25 days after germination in each *HbEBP1* OE line and *Col-0* plants. The total cell number per leaf was calculated by dividing the leaf area by the average cell size. Data were presented as mean ± SEM. Student's *t*-test was carried out for statistical analysis. Significant difference was defined by * *p* < 0.05. (C) Epidermal cells of the seventh leaves from *Col-0*, *OE5*, *OE18* and *OE28* plants.
